# Supplementary material for: Maternal Psychopathological Profile during Childbirth and Neonatal Development during the COVID-19 Pandemic: A Pre-Posttest Study
Source: Behav Sci (Basel). 2023 Jan 18;13(2):80. doi: 10.3390/bs13020080 (PMC9952727; doi:10.3390/bs13020080)
Supplement: Supplementary file 1 [file behavsci-13-00080-s001.zip › behavsci-1934860-supplementary.pdf]

Supplementary Table S1. Socio-demographic, obstetric and clinical data of the pregnant women included in the study according to the psychopathological profile cluster.

	**High psychopathology cluster (N=39)**	**Low psychopathology cluster (N=42)**	**Contrast test**	***p***
Age	31.33 (5.61)	32.76 (5.27)	-1.18	0.24
Civil status			1.56	0.41
Married or cohabiting	99.2	97.6		
Single	5.1	2.4		
Divorced	2.6	0		
Origin			2.86	0.48
Spain	82.1	81		
South-America	17.9	11.9		
Europe	0	4.8		
Morocco	0	2.4		
Primiparous			0.29	0.65
No	51.3	45.2		
Yes	48.7	54.8		
Number of children	1.03 (0.16)	1.12(0.32)	-1.64	0.10
Type of pregnancy			3.35	0.10
Spontaneous	97.4 (88.1)			
Assisted reproductive technology	2.6 (11.9)			
Previous miscarriages	1.72 (0.91)	1.71 (0.97)	0.17	0.98
Work situation			0.42	0.12
Full time	59	64.3		
Part time	10.3	0		
Unemployed	30.8	35.7		
Level of studies			1.89	0.43
Primary	5.1	2.4		
Secondary	59	47.6		
University	35.9	50		
Previous health problems			1.01	0.4
Yes	15.4	24.4		
Belief of having been infected by COVID-19			1.61	0.25
Yes	12.8	23.8		
COVID-19 Positive test				
Yes	7.7	0		0.17
Any positive relatives COVID-19			0.003	0.95
Yes	2.6	2.4		

Note. For continuous variables, the mean and standard deviation (in brackets) and Student's t test statistic are presented. For categorical variables, the

percentage of cases per category and Pearson's $\chi 2$ test statistic (or Fisher's exact test in case of violation of the expected frequencies assumption) are presented.
